# The intestinal microbiota predicts COVID-19 severity and fatality regardless of hospital feeding method

**DOI:** 10.1128/msystems.00310-23

**Published:** 2023-08-07

**Authors:** Vanni Bucci, Doyle V. Ward, Shakti Bhattarai, Mayra Rojas-Correa, Ayan Purkayastha, Devon Holler, Ming Da Qu, William G. Mitchell, Jason Yang, Samuel Fountain, Abigail Zeamer, Catherine S. Forconi, Gavin Fujimori, Boaz Odwar, Caitlin Cawley, Ann M. Moormann, Mireya Wessolossky, Ana Maldonado-Contreras

**Affiliations:** 1 Department of Microbiology and Physiological Systems, University of Massachusetts Chan Medical School, Worcester, Massachusetts, USA; 2 Program of Microbiome Dynamics, University of Massachusetts Chan Medical School, Worcester, Massachusetts, USA; 3 Center for Microbiome Research, University of Massachusetts Chan Medical School, Worcester, Massachusetts, USA; 4 Division of Infectious Diseases and Immunology, University of Massachusetts Chan Medical School, Worcester, Massachusetts, USA; 5 Department of Internal Medicine/Pediatrics, University of Massachusetts Chan Medical School, Worcester, Massachusetts, USA; 6 Department of Medicine - Internal Medicine, University of Massachusetts Chan Medical School, Worcester, Massachusetts, USA; 7 Department of Medicine - Division of Infectious Diseases and Immunology, University of Massachusetts Chan Medical School, Worcester, Massachusetts, USA; University of California Davis, Sacramento, California, USA

**Keywords:** microbiota, COVID-19, predictors, severity, fatality, enteral feeding, diet, random forest classification

## Abstract

SARS-CoV-2-positive patients exhibit gut and oral microbiome dysbiosis, which is associated with various aspects of COVID-19 disease (1–4). Here, we aim to identify gut and oral microbiome markers that predict COVID-19 severity in hospitalized patients, specifically severely ill patients compared to moderately ill ones. Moreover, we investigate whether hospital feeding (solid versus enteral), an important cofounder, influences the microbial composition of hospitalized COVID-19 patients. We used random forest classification machine learning models with interpretable secondary analyses. The gut, but not the oral microbiota, was a robust predictor of both COVID-19-related fatality and severity of hospitalized patients, with a higher predictive value than most clinical variables. In addition, perturbations of the gut microbiota due to enteral feeding did not associate with species that were predictive of COVID-19 severity.

**IMPORTANCE:**

SARS-CoV-2 infection leads to wide-ranging, systemic symptoms with sometimes unpredictable morbidity and mortality. It is increasingly clear that the human microbiome plays an important role in how individuals respond to viral infections. Our study adds to important literature about the associations of gut microbiota and severe COVID-19 illness during the early phase of the pandemic before the availability of vaccines. Increased understanding of the interplay between microbiota and SARS-CoV-2 may lead to innovations in diagnostics, therapies, and clinical predictions.

## OBSERVATION

The microbiota of COVID-19 patients is characterized by a decreased abundance of prototypical anti-inflammatory bacterial species (1 - 5), which may influence overall immune responses or otherwise prime patients for certain risks. However, most microbiome studies in the COVID-19 literature compare patients with moderate or mild symptoms against healthy controls without COVID-19 (1, 6 - 8). The microbiomes of severely ill COVID-19 patients have not been well explored. Additionally, the contributions of enteral feeding (typical in the ICU setting) to COVID-19-related dysbiosis are largely unknown and represent a potential confounder (9, 10). Here, we applied robust and validated machine learning methods with interpretable secondary analyses (11) to better define associations between the microbiota in severe and moderate COVID-19 patients during the early phases of the pandemic.

We enrolled 69 SARS-CoV-2 PCR-positive, hospitalized patients at the University of Massachusetts Medical Center and UMASS Memorial Hospital from 27 April to 10 June 2020. Of the 63 participants ultimately included in our analysis, 22 died of COVID-19 (Table 1). At the time of sample collection, patients requiring >4 L of oxygen were admitted to the intensive care unit due to the severity of their respiratory symptoms; we thus used this as the basis for differentiating moderate versus severe disease. No differences in age, body mass index, gender, race, smoking status, or antibiotic administration during hospitalization were observed between moderately ill and severely ill patients (Table 1). Unsurprisingly, more severely ill patients succumbed to disease and averaged ~6 more days of hospitalization than moderately ill patients. We did not find any significant differences between the groups when considering other symptoms and comorbidity data except that the prevalence of coronary artery disease and hypercholesterolemia was higher in severely ill patients (Table S1).

**TABLE 1:** Characteristics of the COVID-19 hospitalized patients recruited for the study between April and June 2020

	Moderate (*n* = 32)	Severe (*n* = 31)	*P*-value
Demographics			
Age (years)	70.53 + 15.86	70.58 + 14.08	0.8^*a*^
Body mass index (BMI)	29.22 + 9.19	30.58 + 8.82	0.2^*a*^
Female (%)	16 (50%)	10 (30%)	0.2^*b*^
Race			0.2^*c*^
White (%)	24 (75%)	19 (59%)	
Black or African American (%)	4 (12%)	3 (9%)	
Hispanic or Latino (%)	4 (12%)	8 (25%)	
Asian (%)	0 (0%)	2 (6%)	
Smoking status			0.6^*c*^
Current	2 (6%)	4 (12%)	
Former	11 (34%)	11 (34%)	
Never	19 (59%)	16 (50%)	
Antibiotic treatment	26 (81%)	29 (91%)	0.2^*c*^
Days in the hospital	15.5 + 10.39	21.27 + 12.73	**0.045** ^*a*^
Deceased	4 (12.5%)	18 (58%)	**0.0001** ^*c*^

^*a*^ Mann Whitney, unpaired *t*-test.

^*b*^ Fisher exact test.

^*c*^ Chi-square test.

**Fig 1:**
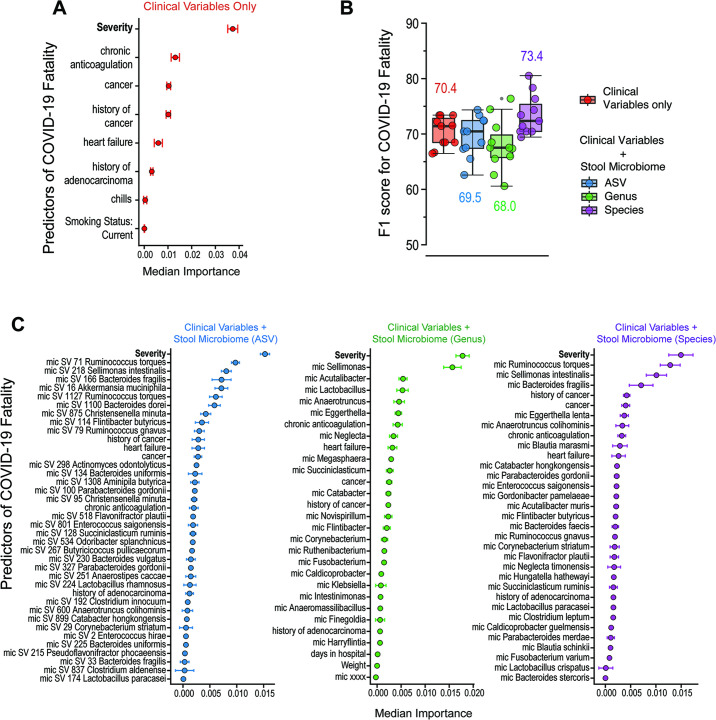
Stool microbiome variables contribute to predictions of COVID-19 fatality. (**A**) Random forest classification modeling using clinical covariates alone identifies severity as the top predictor of fatality. Only clinical variables defined as significant by the Boruta algorithm were included in the model. (**B**) When combined with stool microbiome variables, combined modeling with microbiome abundances classified at the species level improved F1-score compared to clinical variables alone. Boxplots show mean, first, and third quartile scores from 11 modeling runs using the leave-one-out cross-validation method. (**C**) Feature ranking of combined models (clinical plus microbiome variables) identifies severity as the top predictor of fatality regardless of the microbiome abundance classification method. Stool microbiota species outrank most other clinical variables.

Members of the microbiome can predict the severity of inflammatory bowel diseases (12 - 14), the occurrence of colorectal cancer (15, 16), and the immune responses during lung infections with influenza and tuberculosis (17, 18). Thus, to explore markers of dysbiosis in severely ill COVID-19 patients, we collected stool and oral microbiome samples using commercially available kits and sequenced the *16S rRNA* gene using the 341F and 806R universal primers to amplify the V3-V4 region. Sequences were analyzed using DADA2 (19), and species assignment was performed using the same pipeline as previously done by us (18). We used an R-based random forest classification (RFC) algorithm to first predict COVID-19 fatality. To ensure robust modeling, we applied leave-one-out cross-validation and used the Boruta algorithm to perform feature selection to identify relevant clinical variables (i.e., only contributing variables were included in the model). In line with clinical practice and outcomes, our models identified disease severity (defined by the >4 L oxygen requirement) as the main factor in predicting fatality (Fig. 1A). A combination of clinical and stool microbiota variables, specifically when classifying at the species level, was able to predict fatality better than clinical variables alone (F1-score of 73.4 versus 70.4; Fig. 1B). Modeling by feature ranking confirmed that gut bacteria outranked all clinical variables (except for severity) in predictive importance for COVID-19 fatality (Fig. 1C). We also applied the Stable and Interpretable RUIe Set (SIRUS), an interpretable rules algorithm (11), and obtained higher predictability of COVID-19 fatality by microbiome composition (63% probability of death) than clinical variables alone. The oral microbiota poorly predicted COVID-19 fatality in this cohort of hospitalized patients (Fig. S1).

**Fig 2:**
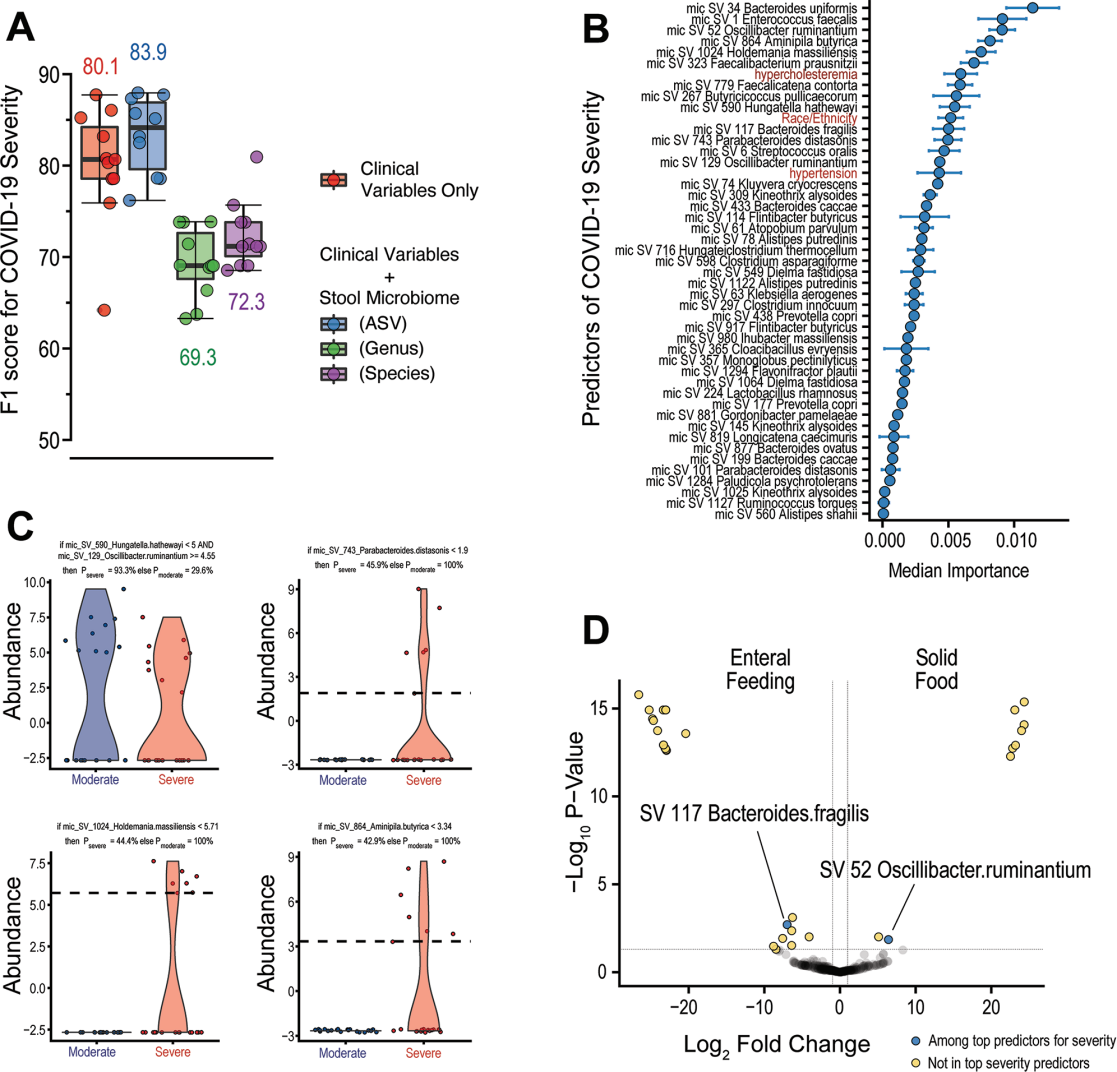
Stool microbiota can help predict COVID-19 severity and are independent of hospital feeding methods. (**A**) Combined random forest classification modeling of clinical variables and stool microbiome abundances classified at the ASV level improved the prediction of COVID-19 severity in our cohort. Boxplots show mean, first, and third quartile scores from 11 modeling runs using the leave-one-out cross-validation method. (**B**) Feature ranking of the combined model using ASV classification shows that stool microbiota species outrank most clinical variables in predictive importance. (**C**) SIRUS identified abundance changes in certain stool species that were predictive of severe or moderate disease. (**D**) Analysis of species correlated with enteral feeding or solid hospital food diet identified only two species that were among the top bacteria predictors for severity.

Given the impact of severity on predicting COVID-19 fatality and indications of a role for stool microbiota, we next explored how gut microbiota may contribute to severity. We thus applied our RFC modeling pipeline to target outcomes of patients requiring <4 L (moderate disease) or >4 L of oxygen (severe disease). A combination of clinical and stool microbiome variables, specifically when classifying at the amplicon sequence variant (ASV) level, was able to predict COVID-19 severity better than clinical variables alone (F1-score of 83.9 versus 80.1; Fig. 2A). Feature ranking on model results revealed members of the classes Bacteroidia (*Bacteroides uniformis* and *B. fragilis*), Clostridia (*Oscillibacter ruminatium, Aminipila butyrica, Faecalibacterium prausnitzii, Faecalicatena contorta,* and *Butyricicoccus pullicaecorum*), and Bacilli (*Haloimpatiens massiliensis* and *Enterococcus faecalis*) along with hypercholesteremia are among the top 10 variables that predicted COVID-19 severity (Fig. 2B). Finally, SIRUS identified that an increased and decreased abundance of two Clostridia species (*O. ruminantium* and *Hungatella hathewayi*, respectively) are associated with a high probability (>90%) of severe disease, while patients with moderate disease exhibited a decreased abundance of Bacteroidia (*Parabacteroides distasonis),* Bacilli (*Haloimpatiens massiliensis*), and Clostridia (*Aminipila butyrica*) along with normal cholesterolemia (Fig. 2C). Results of fatality and severity models using a combination of the oral microbiome and clinical variables were less predictive than clinical variables alone (Fig. S1).

As severely ill patients admitted to the ICU were also more likely to be fed enterally, we felt it important to explore the associations between severity, enteral feeding, and gut microbiota. Ninety percent of patients with moderate symptoms were fed a solid hospital diet, whereas 63% of patients with severe symptoms received enteral feeding at the time of sample collection. Interestingly, the top bacterial species predictors of COVID-19 severity differed greatly from those linked to differences in diet. Only two species were found to be predictors of both COVID-19 severity and enteral feeding, namely, *O*. *ruminantium* and *B*. *fragilis* (Fig. 2D). While *O. ruminantium* and *B. fragilis* ranked 3rd and 12th among predictors, respectively, these two species may simply predict dietary differences rather than disease severity; most species found to be depleted or overly abundant between enterally fed patients and patients on solid food were not among the top predictors for COVID-19 severity (Fig. 2B; Fig. S2). Notably, patients receiving enteral feedings were mostly depleted of Clostridia species. Together, our findings indicate that differences in stool microbiota associated with COVID-19 severity are independent of differences in hospital diet.

We observed distinct bacterial markers within the intestinal microbiota that predicted COVID-19 severity, the main clinical risk factor for fatality. The predictive power of the gut microbiota outranked clinical variables in our cohort, suggesting a pathophysiologic role for gut microbiota in COVID-19. The bacteria identified as predictors in this study are comparable to those found in previous studies comparing severe COVID-19 patients and healthy controls (1, 6 - 20 - 20). Further mechanistic studies are needed to understand whether the gut microbiota affects the pathophysiology of COVID-19. While the results of our study are limited by one-time sample collection and small sample size, our machine learning models were still able to provide meaningful associations, considering the effects of enteral feeding. Additionally, these data were gathered in early 2020 and, thus, offer insight into possible biologic links between the gut microbiota and COVID-19 before the availability of vaccines. Finally, we acknowledge that our results could also be explained by the fact that in systemically ischemic patients, there could be a loss of gut barrier integrity and, thus, microbiome-associated dysbiosis (21).

## Data Availability

16S sequencing data used in this study are available for download through the NCBI BioProject (https://www.ncbi.nlm.nih.gov/bioproject) under NCBI BioProject number PRJNA868760 (“The Intestinal and Oral Microbiomes of SARS-CoV-2 PCR positive, hospitalized patients”). R-code used to generate models is publicly available on Github.

## SUPPLEMENTAL MATERIAL

Supplemental Tables and Figures msystems.00310-23-s0001.pdf Figures S1 and S2 and Tables S1 and S2.

Supplemental Figure Legends msystems.00310-23-s0002.docx Legends to Fig. S1 and S2.

ASM does not own the copyrights to Supplemental Material that may be linked to, or accessed through, an article. The authors have granted ASM a non-exclusive, world-wide license to publish the Supplemental Material files. Please contact the corresponding author directly for reuse.
