# Supplementary material for: The intestinal microbiota predicts COVID-19 severity and fatality regardless of hospital feeding method
Source: mSystems. 2023 Aug 7;8(4):e00310-23. doi: 10.1128/msystems.00310-23 (PMC10469851; doi:10.1128/msystems.00310-23)
Supplement: Supplemental Tables and Figures — Figures S1 and S2 and Tables S1 and S2. [file msystems.00310-23-s0001.pdf]

Supplementary Table 1. List of clinical variables included in the analyses. Of all the clinical variables included in the models, only coronary artery disease and hypercholesterolemia were statistically different between moderate and severe COVID-19 patients.

**Clinical variables**<br>(Obtained by review of medical records)	**Moderate (n=32)**	**Severe (n=31)**	**Mann Whitney tests (p value)**
Symptoms			
Abdominal pain	1	1	>0.999999
Chest pain	2	3	0.671867
Chills	5	6	0.74996
Cough	12	12	>0.999999
Diarrhea	3	4	0.707846
Difficulty breathing	16	22	0.12349
Dizziness	1	0	>0.999999
Dyspnea	14	19	0.210129
Fever	11	17	0.131294
Hemoptysis	0	1	0.492063
Hypotension	4	2	0.671867
Hypoxic respiratory distress	18	25	0.057677
Shortness of breath	16	21	0.202826
Sore throat	1	0	>0.999999
Tachycardia	9	11	0.595035
Tachypnea	11	17	0.131294
Vomiting	4	2	0.671867
Anemia	3	5	0.474123
Co-morbidities			
Asthma	7	2	0.147716
Bradycardia	2	0	0.492063
Cardiomyopathy	3	2	>0.999999
Cancer	6	8	0.556127
Cerebellar atrophy	0	0	>0.999999
Cerebral palsy	1	0	>0.999999
Chronic fibrillation	6	8	0.556127
Chronic anticoagulation	3	6	0.302018
Heart failure	8	12	0.286909
Kidney disease	7	8	0.773534
Chronic respiratory failure	2	7	0.081587
Thrombosis	4	1	0.354671

Cirrhosis	1	1	>0.999999
Congestive heart failure	7	12	0.176864
COPD	3	7	0.183638
**Coronary artery disease**	**2**	**8**	**0.043318**
Emphysema	0	2	0.238095
End stage renal disease	2	5	0.248622
Epilepsy	2	4	0.425766
Goodpasture syndrome	1	0	>0.999999
Heart attack	0	1	0.492063
Heart failure	8	13	0.187691
Hepatic encephalopathy	1	0	>0.999999
History (hx) adenocarcinoma	1	3	0.354671
History (hx) aortic aneurysm	1	0	>0.999999
History (hx) cancer	6	8	0.556127
**Hypercholesteremia**	**1**	**11**	**0.001153**
Hyperglycemia	13	15	0.61593
Hyperlipidemia	16	13	0.620886
Hypertension	23	26	0.36492
Hypotension	0	1	0.492063
Interstitial lung disease	0	1	0.492063
Lymphoma	1	0	>0.999999
Multiple myeloma	1	1	>0.999999
Nephrectomy	0	1	0.492063
Parkinson’s	0	1	0.492063
Renal disease	7	8	0.773534
Renal failure	2	5	0.256519
Stage 3 chronic kidney disease	4	4	>0.999999
Stage 4 adenocarcinoma	1	1	>0.999999
Stage 5 end-stage renal disease	2	5	0.256519
Type 1 diabetes	0	1	0.492063
Type 2 diabetes	13	15	0.61593

Supplementary Table 2. Performance metrics of different Random Forest Classification Models predicting COVID-19 severity (moderate *vs.* severe). SEN=Sensitivity, SPEC=Specificity, PPV=Positive Predicted Values, NPV=Negative Predicted Values, PREC=Precision, REC=Recall, F1=F1 Score.

MODEL	**SEN**	**SPEC**	**PPV**	**NPV**	**PREC**	**REC**	**F1**
CC	0.68	0.68	0.68	0.68	0.68	0.68	0.68
STL	0.86	0.85	0.86	0.85	0.86	0.86	0.86
TNG	0.72	0.87	0.84	0.76	0.84	0.72	0.78
CC + STL	1.00	0.80	0.85	1.00	0.85	1.00	0.92
CC + TNG	0.76	0.90	0.88	0.79	0.88	0.76	0.81

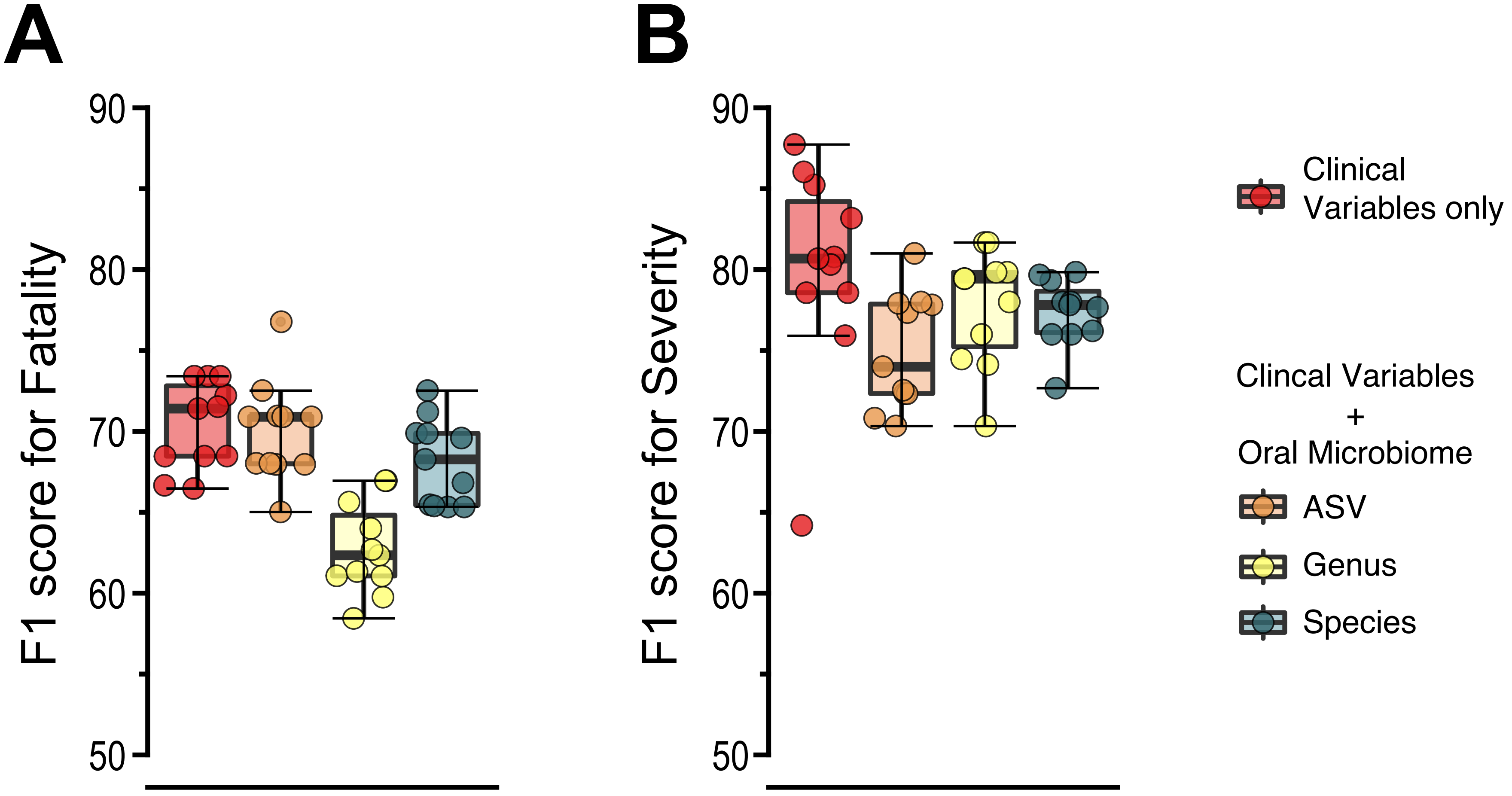
A
B
F1 score for Fatality
F1 score for Severity
90
80
70
60
50
Clinical
Variables only
Clincal Variables
+
Oral Microbiome
ASV
Genus
Species

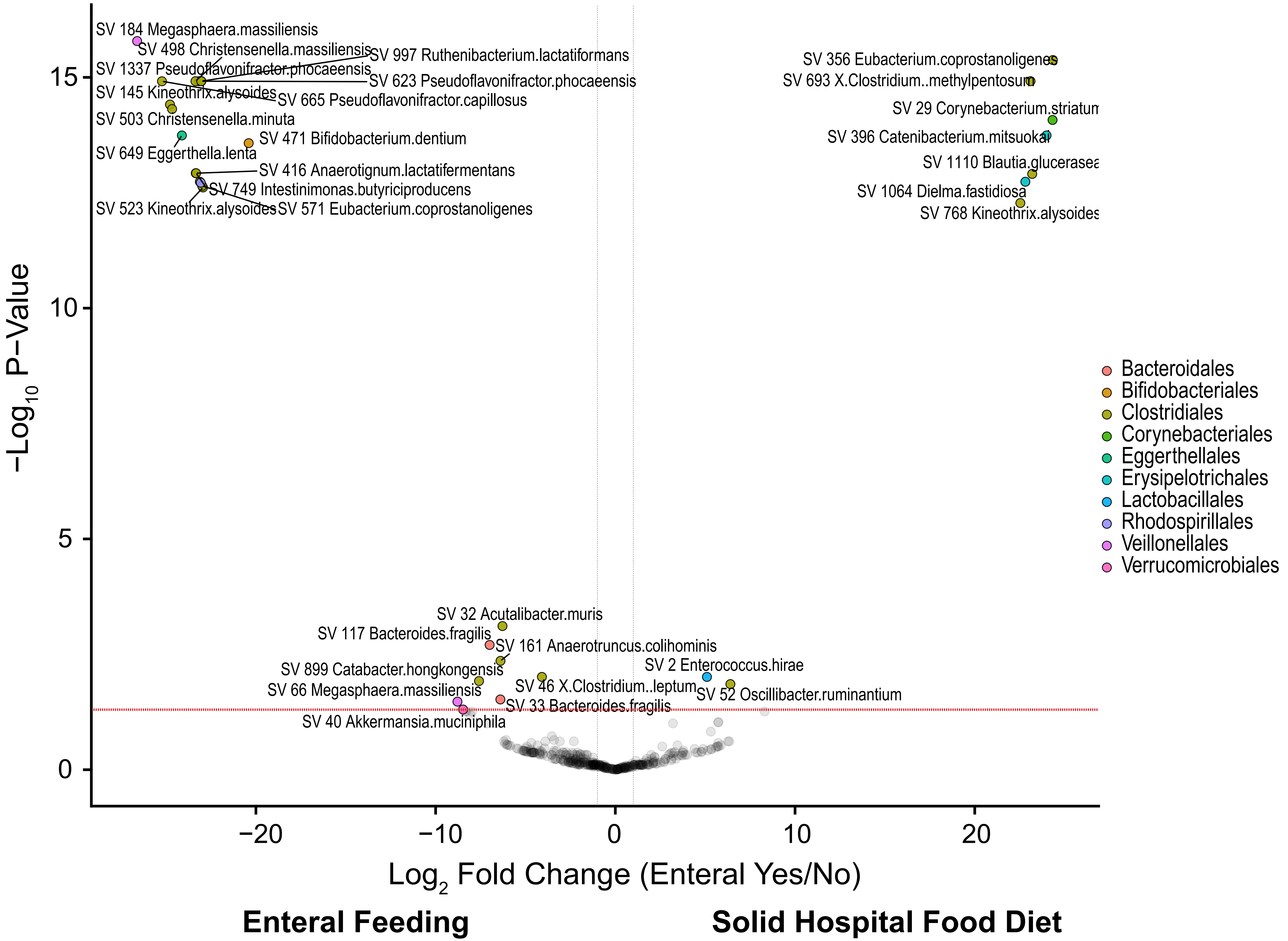

SV 184 Megasphaera.massiliensis
SV 498 Christensenella.massiliensis
SV 997 Ruthenibacterium.lactatiformans
SV 1337 Pseudoflavonifractor.phocaeensis
SV 623 Pseudoflavonifractor.phocaeensis
SV 145 Kineothrix.alysoides
SV 665 Pseudoflavonifractor.capillosus
SV 503 Christensenella.minuta
SV 471 Bifidobacterium.dentium
SV 649 Eggerthella.lenta
SV 416 Anaerotignum.lactatifermentans
SV 749 Intestinimonas.butyriciproducens
SV 523 Kineothrix.alysoides
SV 571 Eubacterium.coprostanoligenes
SV 356 Eubacterium.coprostanoligenes
SV 693 X.Clostridium..methylpentosum
SV 29 Corynebacterium.striatum
SV 396 Catenibacterium.mitsuokai
SV 1110 Blautia.glucerasea
SV 1064 Dielma.fastidiosa
SV 768 Kineothrix.alysoides
SV 32 Acutalibacter.muris
SV 117 Bacteroides.fragilis
SV 161 Anaerotruncus.colihominis
SV 2 Enterococcus.hirae
SV 899 Catabacter.hongkongensis
SV 46 X.Clostridium..leptum
SV 52 Oscillibacter.ruminantium
SV 66 Megasphaera.massiliensis
SV 33 Bacteroides.fragilis
SV 40 Akkermansia.muciniphila
15
10
5
0
−20
−10
0
10
20
$-Log_{10}$ P−Value
Log_2 Fold Change (Enteral Yes/No)
Bacteroidales
Bifidobacteriales
Clostridiales
Corynebacteriales
Eggerthellales
Erysipelotrichales
Lactobacillales
Rhodospirillales
Veillonellales
Verrucomicrobiales
Enteral Feeding
Solid Hospital Food Diet
