# Supplementary material for: The intestinal microbiota predicts COVID-19 severity and fatality regardless of hospital feeding method
Source: mSystems. 2023 Aug 7;8(4):e00310-23. doi: 10.1128/msystems.00310-23 (PMC10469851; doi:10.1128/msystems.00310-23)
Supplement: Supplemental Figure Legends — Legends to Fig. S1 and S2. [file msystems.00310-23-s0002.docx]

**Supplemental Figure 1: The oral microbiome did not contribute to predictions of COVID-19 fatality or severity.** Combined Random Forest classification modeling of clinical variables and oral microbiome abundances classified at the species, genus, or ASV levels did not improved prediction of COVID-19 fatality (A) or severity (B) in our cohort. Boxplots show mean, 1^st^ and 3^rd^ quartile scores from 11 modeling runs using the leave-one-out cross validation method.

**Supplemental Figure 2: Deseq2 analysis of microbiota differences between hospitalized COVID-19 patients on enteral feeding (left) or solid diet (right).** Individual species above threshold are labeled.
